# Ferric carboxymaltose in patients with restless legs syndrome and nonanemic iron deficiency: A randomized trial

**DOI:** 10.1002/mds.27040

**Published:** 2017-06-23

**Authors:** Claudia Trenkwalder, Juliane Winkelmann, Wolfgang Oertel, Garth Virgin, Bernard Roubert, Anna Mezzacasa, on behalf of the FCM‐RLS Study Investigators

**Affiliations:** ^1^ Department of Neurosurgery University Medical Center Göttingen Germany; ^2^ Paracelsus‐Elena Klinik Kassel Germany; ^3^ Neurologische Klinik und Poliklinik Klinikum rechts der Isar, Technische Universität München Munich Germany; ^4^ Institut für Neurogenomik Helmholtz Zentrum München Munich Germany; ^5^ Munich Cluster for Systems Neurology (SyNergy) Munich Germany; ^6^ Department of Neurology Philipps‐University Marburg Marburg Germany; ^7^ Vifor Pharma Glattbrugg Switzerland

**Keywords:** restless legs syndrome, intravenous iron, iron deficiency, ferric carboxymaltose, clinical trial

## Abstract

**Background**: Compromised iron status is important in restless legs syndrome pathophysiology. We compared the efficacy and tolerability of ferric carboxymaltose (single intravenous dose) versus placebo for restless legs syndrome treatment in iron‐deficient nonanemic patients.

**Methods**: Patients with moderate to severe restless legs syndrome and serum ferritin < 75 μg/L (or serum ferritin 75‐300 μg/L and transferrin saturation < 20%) were randomized to ferric carboxymaltose (1000 mg iron) or placebo. Mean change difference between ferric carboxymaltose and placebo in International Restless Legs Syndrome Severity Scale score from baseline to week 4 was the primary end point; week 12 was a secondary end point.

**Results**: Ferric carboxymaltose treatment (n = 59) led to nonsignificant improvement over placebo (n = 51) in International Restless Legs Syndrome Severity Scale score at week 4 (difference [95% confidence interval], ‐2.5 [‐5.93 to 1.02], *P* = 0.163), reaching significance by week 12 (‐4.66 [‐8.59 to ‐0.73], *P* = 0.021).

**Conclusions**: In patients who responded to treatment, ferric carboxymaltose may require more time to stabilize restless legs syndrome than previously assumed. © 2017 The Authors. Movement Disorders published by Wiley Periodicals, Inc. on behalf of International Parkinson and Movement Disorder Society.

Restless legs syndrome (RLS) pathophysiology is still only partly understood, with the involvement of the dopaminergic system still under debate.^1^ However, genetic studies thus far have failed to link dopamine pathology to RLS.^2^ Compromised iron status is an important disorder, possibly contributing to the cause of RLS.^3^ However, the causal relationship still remains unclear, and there is much debate about how to adequately assess iron status in RLS patients.^4^, ^5^, ^6^, ^7^, ^8^ The potential relationship between low iron status and RLS has led to increased interest in the use of iron replacement therapy to treat this disorder.^1^ Study results have varied, most likely because of the different iron formulations and dosages used, as well as study design. Although some previous studies excluded anemic patients,^7^ all studies to date have included patients of varied iron status, but have not provided clear evidence about which RLS patients might benefit most from iron treatment.^6^, ^7^, ^8^, ^9^, ^10^, ^11^

Previously, a single treatment of ferric carboxymaltose (FCM) provided significant and prolonged reduction (>24 weeks) in RLS symptoms, with minimal adverse events (AEs) in 2 small cohorts of patients.^6^, ^12^ FCM has been shown to also provide significant reduction in RLS symptoms in pregnant women.^11^ However, these studies included patients with a broad range of serum ferritin levels, making it difficult to establish any relationship between pretreatment serum ferritin level and response to therapy.^6^, ^12^

This study compared the efficacy and tolerability of a single 1000‐mg infusion of intravenous iron as FCM versus placebo, as a treatment for RLS in patients with impaired iron status.

## Methods

### Patients

Patients aged ≥18 years weighing ≥50 kg with moderate to severe RLS (International RLS Severity Scale [IRLS] total score ≥15), normal hemoglobin levels (women, ≥11.5 g/dL; men, ≥12.5 g/dL), and serum ferritin <75 μg/L were eligible for this study (patients were also included if serum ferritin was between 75 and 300 μg/L and transferrin saturation [TSAT] was <20%). All patients provided appropriate written informed consent. Further details can be found in the Supplementary Materials.

### Study Design

This trial was a prospective phase 4 patient‐ and assessor‐blind (the study nurse who administered the treatment was not blinded), placebo‐controlled 12‐week multicenter study (EudraCT number: 2013‐000574‐30). Eligible patients were randomized before the start of treatment to either a single intravenous dose of 1000 mg iron as ferric carboxymaltose or placebo by drip infusion over 15 ± 2 minutes on day 1 (see Supplementary Material). This study was conducted in accordance with the principles of the Declaration of Helsinki and Good Clinical Practice guidelines. More details can be found in the Supplementary Materials.

### Study Objectives and End Points

The primary objective of this study was to compare the efficacy of FCM versus placebo as treatment for RLS in iron‐deficient nonanemic patients. The primary efficacy end point was the difference in mean change in IRLS score from baseline to week 4 between FCM therapy and placebo; change from baseline to week 12 was a secondary efficacy end point. Mean change in serum ferritin and TSAT levels and tolerability were monitored throughout. Other secondary efficacy end points are described in the Supplementary Materials.

### Assessments

Assessments were performed at baseline, weeks 1, 4, 8, and 12, and at the end of study (EOS; ie, week 12 or following earlier termination), unless otherwise stated. Efficacy was primarily assessed with the patient‐based IRLS scale. Secondary assessments included the Clinical Global Impression scale items‐1 and ‐2, the Patient Global Impression of Improvement Index, RLS‐6 scale, and quality‐of‐life (QoL) questionnaire. Safety was assessed by monitoring the occurrence or increase in severity of AEs throughout the study and up to 30 days after administration of treatment or at last study visit, whichever was longer. More details can be found in the Supplementary Materials.

### Statistical Analyses

The sample size (55 per treatment arm) was calculated assuming an improvement of IRLS total score of at least 4.5 in the FCM group compared with placebo at week 4,^6^ using a standard deviation of 8 points, 5% 2‐sided type I error, and 80% power. All efficacy analyses were conducted on the full analysis set (FAS), and safety and tolerability analyses were conducted on the safety set. For further details, please see Supplementary Materials.

## Results

### Patient Population

A total of 110 patients (mean ± SD age, 54.1 ± 15.8 years; 82% female; 49% previously received dopaminergic treatment; Table 1) from 13 sites (Finland, 3 [n =36]; Germany, 8 [n = 70]; Switzerland: 2 [n = 4]) were randomized to receive a single intravenous dose of FCM (n=59) or placebo (n=51) and included in the FAS (Suppl. Fig. 1). Patient demographics and baseline characteristics were similar between treatment arms (Table 1), and 87 patients (79%) completed the study.

**Table 1:** Patient demographics and baseline characteristics

Variable^a^	Ferric carboxymaltose (n = 59)	Placebo (n = 51)
Age, years	53.0 (15.7)	55.5 (15.9)
Age category, n (%)		
<65 years	41 (69.5)	35 (68.6)
65‐74 years	15 (25.4)	11 (21.6)
75‐84 years	3 (5.1)	5 (9.8)
Female, n (%)	48 (81.4)	42 (82.4)
Weight, kg	73.88 (16.58)	73.89 (13.10)
Height, cm	166.1 (8.64)	167.0 (7.71)
BMI, kg/m^2^	26.77 (5.70)	26.46 (4.09)
Previous dopaminergic treatment, n (%)	31 (53.4)	23 (44.2)
Serum ferritin, μg/L	41.93 (34.55)	48.85 (45.95)
TSAT, %	18.49 (7.88)	21.14 (9.19)
IRLS total score	25.9 (5.65)	26.0 (5.78)
CGI‐Item 1 severity score	4.8 (0.87)	4.7 (0.74)
RLS‐6		
Sleep satisfaction	7.2 (2.53)	7.2 (2.52)
Severity when falling asleep	6.8 (2.66)	6.2 (3.03)
Severity during the night	5.9 (3.06)	5.7 (3.18)
Severity during the day, at rest	4.5 (3.06)	4.3 (2.88)
Severity during the day, active	1.8 (2.30)	2.5 (2.69)
Daytime tiredness	5.9 (2.60)	5.6 (3.07)

a Mean (standard deviation) unless otherwise stated.

BMI, body mass index; CGI, Clinical Global Impressions; IRLS, International Restless Legs Scale; RLS‐6, Restless Legs Syndrome‐6; TSAT, transferrin saturation.

### Efficacy

**Figure 1:**
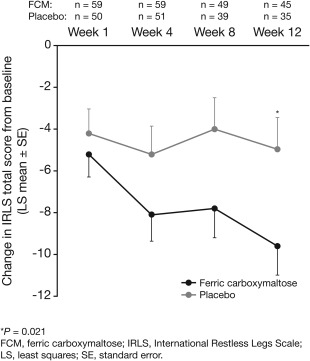
Changes over time in IRLS total score.

The primary end point was not met after 4 weeks, with a change in IRLS (LS mean, last observation carried forward [LOCF]) of ‐7.7 (standard error [SE] = 1.1) for patients treated with FCM and ‐5.2 (SE = 1.2) for those treated with placebo (treatment difference, ‐2.9; 95% confidence interval [CI], ‐5.93 to 1.02; *P* = 0.163; Fig. 1). However, the mean decrease in IRLS total score from baseline was greater with FCM versus placebo at all study visits and led to a significant improvement by week 12 (FCM, ‐9.6 ± 1.4; placebo, ‐5.0 ± 1.5; treatment difference, ‐4.66 [95%CI, ‐8.59 to ‐0.73]; *P* = 0.021; Fig. 1). Results from most other secondary end points were also significantly in favor of FCM (for other RLS symptom severity, QoL, and sleep results, see Supplementary Materials).

### Patient Iron Status

Changes in serum ferritin and TSAT levels from baseline were significantly increased with FCM treatment versus placebo throughout the study (*P* < 0.001). There was a significant correlation between baseline TSAT level and IRLS improvement with FCM treatment at week 4 (*r* = 0.37; *P* = 0.006) and EOS (*r* = 0.28; *P* = 0.031). There was no correlation between baseline serum ferritin level or increased iron parameter level and IRLS improvement.

### Tolerability

Overall, 33 treatment‐emergent AEs (TEAEs) were reported in 23 patients (FCM, 16 patients, 27.1%; placebo, 7 patients, 13.7%; Suppl. Table 3), the majority of which were mild or moderate in severity. Severe TEAEs were experienced by 4 patients in the FCM group and 2 with placebo. Serious AEs were reported in 2 patients, 1 from each treatment group, which led to their withdrawal from the study. No hypersensitivity reactions, anaphylactoid reactions, or deaths were reported. For additional details, please refer to the Supplementary Materials.

## Discussion

The results of this study in iron‐deficient nonanemic patients showed that a single 1000‐mg dose of intravenous iron as FCM provides nonsignificant differences in IRLS after 4 weeks of treatment (primary end point) compared with placebo; however, FCM provides a significant and clinically meaningful difference^13^, ^14^ after 12 weeks (treatment difference, ‐4.66; *P* = 0.021). Unlike other treatments, which may be administered several times over the trial period,^6^, ^8^ FCM was administered as a single intravenous dose. This may have increased the likelihood of withdrawal if patients were administered placebo treatment or possibly had a noniron‐sensitive RLS phenotype and could not tolerate RLS symptoms over time. Nevertheless, patients who did respond to FCM treatment achieved the greatest improvements in IRLS scores at varying times, suggesting either an early or late responder in iron‐sensitive IRLS phenotypes. This time‐delayed response to FCM in the current study is different compared with trials examining dopaminergic or opioidergic agents. In support of the IRLS assessment, most other RLS symptom severity and QoL scores were also significantly improved with FCM treatment by week 12.

RLS symptom severity assessments such as IRLS, CGI, and even RLS‐6 are gold standard methods for measuring RLS treatment efficacy.^15^, ^16^, ^17^ IRLS findings observed in the current study are similar to those seen in previous randomized, placebo‐controlled trials. Allen et al reported a significant 5‐point difference in mean IRLS total score between FCM and placebo at week 4 (*P* = 0.049).^6^ Similarly, Cho et al also reported a significant 12‐point difference in IRLS score between FCM and placebo at week 6 (*P* = 0.03).^12^ However, unlike the current study, there were no serum ferritin threshold inclusion criteria in either study, making it difficult to determine which patients would benefit from treatment.^6^, ^12^

Severity of RLS is worse when at rest during the day and evening,^17^ which is significantly alleviated with FCM treatment, as reflected in RLS‐6 scores for both at rest during the day, and in the evening. The nonsignificant improvements in RLS‐6 for “severity during the day when active” further support the suggestion that only RLS‐characteristic symptoms were improved with FCM.

Although the causal relationship between compromised iron status and RLS remains unclear, our findings support a role for iron replacement therapy in the treatment of RLS. Iron has important roles in oxygen metabolism and energy capacity,^18^ and RLS is often associated with conditions involving hypoxia.^19^, ^20^, ^21^ Notably, baseline TSAT levels correlated significantly with improvement in IRLS scores at week 4 and EOS. Patients with a lower baseline TSAT value appeared to have a greater improvement in IRLS score, suggesting that patients who are more iron deficient may benefit from FCM treatment. The maintenance of higher iron levels with FCM may boost oxidative metabolism, resulting in the significant improvements observed in this study.

## Conclusions

Although the primary efficacy end point at week 4 was not met, a single 1000‐mg dose of iron as FCM could provide significant improvements in RLS symptoms after 12 weeks in this iron‐deficient nonanemic RLS population. The mechanisms underlying this effect are still not fully understood; for example, early versus late responders, or lack of response to iron treatment, could reflect a phenotype of patients with causes of RLS independent of iron status. Furthermore, large‐scale studies are needed to identify subgroups of patients who will benefit most from FCM treatment.

## Authors' Roles

(1) Research Project: A. Conception, B. Organization, C. Execution; (2) Statistical Analysis: A. Design, B. Execution, C. Review and critique; (3) Manuscript Preparation: A, Writing, B. Review, and C. Critique

C.T.: 1A, 1B, 1C, 3A

W.O.: 1A, 1C

J.W.: 1A, 1C

G.V.: 1A, 1B, 2A

A.M.: 1A, 1B, 2A

B.R.: 2A, 2C

All: 2C, 3B, 3C

## Financial Disclosures of All Authors

Claudia Trenkwalder has received advisory board fees from Britannia Pharmaceuticals, Mundipharma, Novartis, and UCB, lecture payments from AbbVie, AstraZeneca, Desitin, Mundipharma, and UCB, and royalties from Schattauer Verlag. Juliane Winkelmann has received financial support from DFG, DFG Synergy Excellence Cluster, Else Kröner Foundation, and German Research Foundation. Wolfgang Oertel is treasurer of the European Brain Council and European RLS Study group, speaker of the German Parkinson Study group, member of the Scientific Panel for Health (SPH – DGXII, EU), stock owner at BiogenIdec, Medigene, Merck Darmstadt, and Roche, consultant for Adamas, and Novartis, on the advisory board for Adamas, Bristol Myers Squibb, Neuropore, Novartis, Roche, and UCB, and received honoraria or grants from AbbVie, Desitin, GE Health, Mundipharma, Novartis, UCB, Charitable Hertie Foundation, Germany Federal Ministry of Education and Research, German Research Foundation, Michael J. Fox Foundation, Internationaal Parkinson Fonds, and Novartis Pharma Germany. Garth Virgin, Bernard Roubert, and Anna Mezzacasa are employees of Vifor Pharma.

## Supporting information

Additional Supporting Information may be found in the online version of this article at the publisher's website.

Supplementary Information Figure 1.

Supplementary Information Figure 2.

Supplementary Information Tables.

Supplementary Information
