# Supplementary figures and images for: Ferric carboxymaltose in patients with restless legs syndrome and nonanemic iron deficiency: A randomized trial
Source: Mov Disord. 2017 Jun 23;32(10):1478–82. doi: 10.1002/mds.27040 (PMC5655783; doi:10.1002/mds.27040)

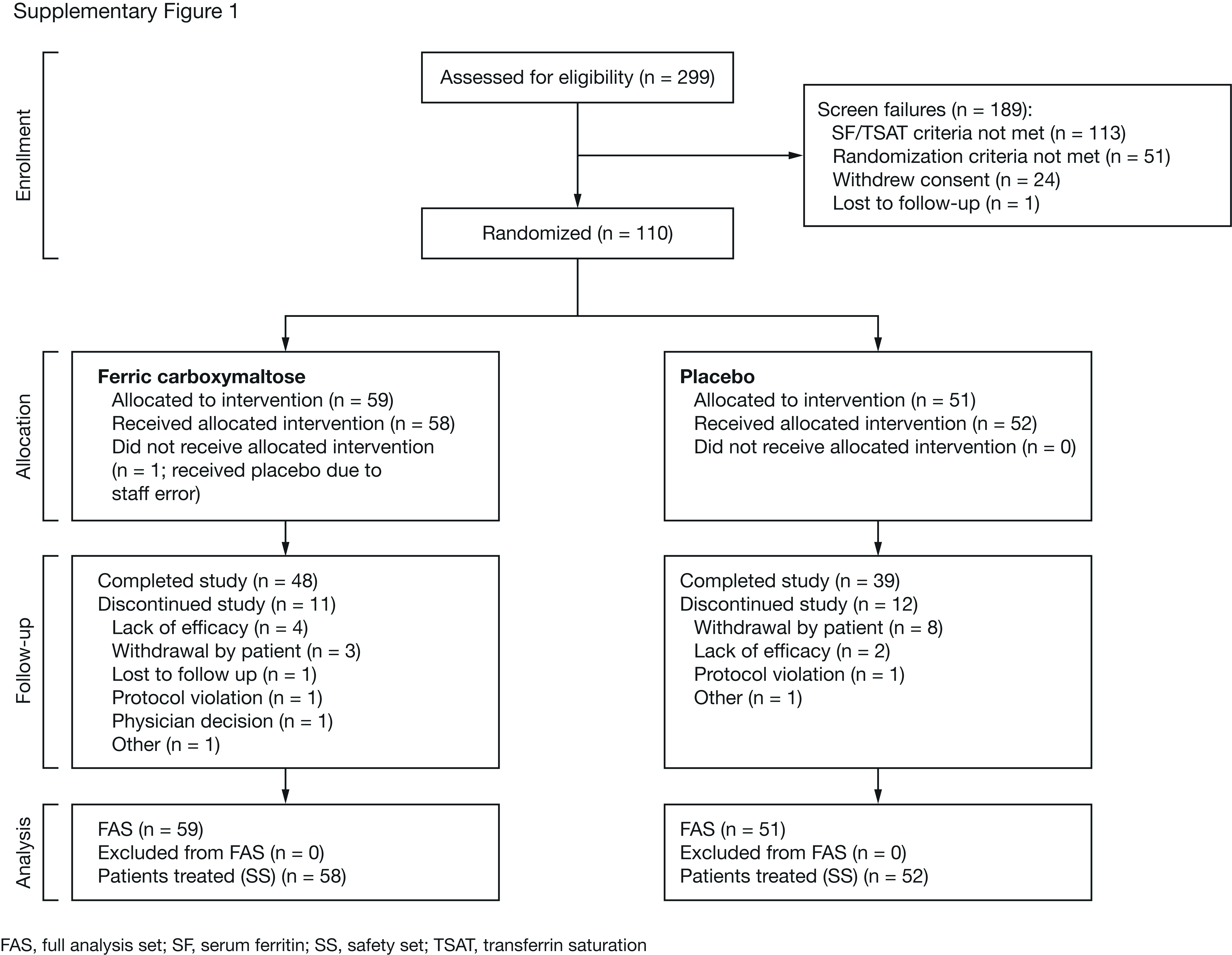

Supplement: Supplementary file 1 — Supplementary Information Figure 1. [file MDS-32-1478-s001.tif]

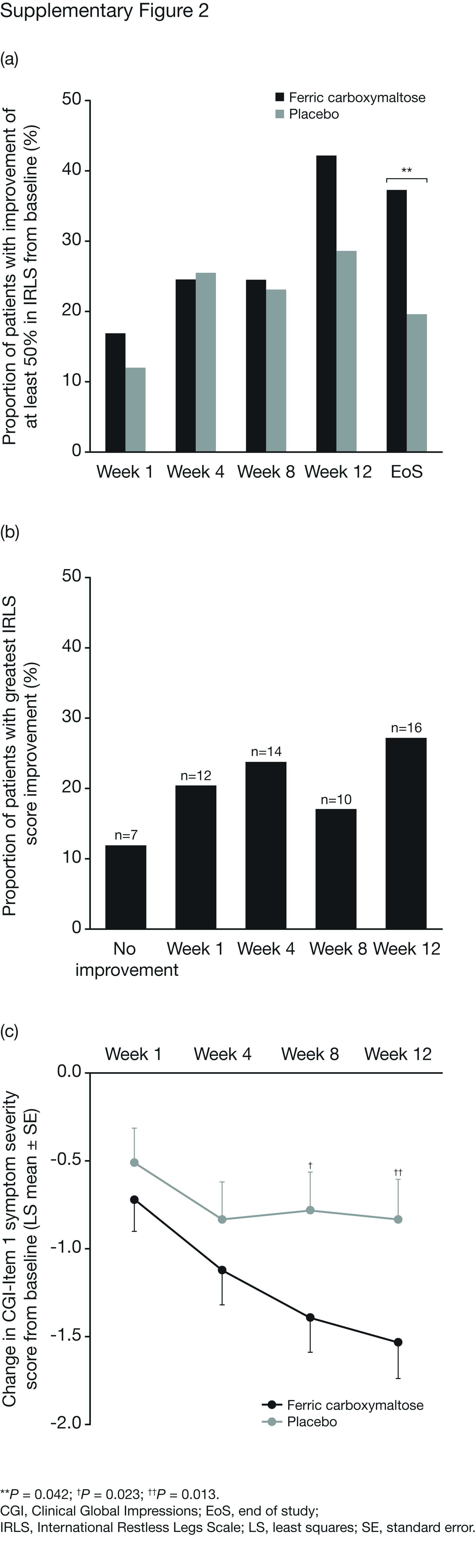

Supplement: Supplementary file 2 — Supplementary Information Figure 2. [file MDS-32-1478-s002.tif]
