# Supplementary material for: Ferric carboxymaltose in patients with restless legs syndrome and nonanemic iron deficiency: A randomized trial
Source: Mov Disord. 2017 Jun 23;32(10):1478–82. doi: 10.1002/mds.27040 (PMC5655783; doi:10.1002/mds.27040)
Supplement: Supplementary file 3 — Supplementary Information Tables. [file MDS-32-1478-s003.docx]

**SUPPLEMENTARY TABLE 1. Medications taken prior to trial initiation
(≥2% incidence in either treatment arm)**

| **Prior medication, n (%)*** | **Ferric carboxymaltose**  **(n = 58)** | **Placebo  (n = 52)** |
| --- | --- | --- |
| - Any prior medication | 42 (72) | 31 (60) |
| - Anti-Parkinson drugs | 31 (53) | 23 (44) |
| Dopamine agonists | 23 (40) | 14 (27) |
| Pramipexole | 22 (38) | 11 (21) |
| Rotigotine | 3 (5) | 3 (6) |
| Ropinirole | 0 | 3 (6) |
| Dopa and Dopa derivatives | 10 (17) | 15 (29) |
| Madopar | 9 (16) | 13 (25) |
| Levodopa | 1 (2) | 2 (4) |
| Psycholeptics | 7 (12) | 3 (6) |
| Benzodiazepine derivatives | 4 (7) | 2 (4) |
| Oxazepam | 2 (3) | 0 |
| Other hypnotics and sedatives | 3 (5) | 0 |
| Doxepin | 3 (5) | 0 |
| Benzodiazepine-related drugs | 2 (3) | 1 (2) |
| Antiepileptics | 2 (3) | 1 (2) |
| Anti-inflammatory and antirheumatic products | 2 (3) | 1 (2) |
| Psychoanaleptics | 2 (3) | 1 (2) |
| Antianemic preparations | 0 | 3 (6) |
| Iron | 0 | 3 (6) |

*Coded using the WHO Drug Dictionary version 2013.

**SUPPLEMENTARY TABLE 2. Symptom severity according to Clinical Global Impressions-Item 2, Patient Global Impressions of Improvement, and Restless Legs Syndrome-6 scores**

| **Proportion of patients with a rating of “much improved” in CGI-Item 2 and “very much improved” in PGI-I** | | | | | | | |
| --- | --- | --- | --- | --- | --- | --- | --- |
|  | **FCM  (n = 59)**  **n (%)** | **Placebo  (n = 51)**  **n (%)** | | | **Odds ratio**  **(95% CI)** | | ***P* value** |
| Week 1 (CGI only) | 17 (28.8) | 8 (16.3) | | | 2.1 (0.8, 5.4) | | 0.127 |
| Week 4 | 23 (43.4) | 7 (14.9) | | | 4.7 (1.8, 12.7) | | 0.002 |
| Week 8 (CGI only) | 28 (57.1) | 10 (25.6) | | | 3.0 (1.1, 8.5) | | 0.003 |
| Week 12 | 19 (42.2) | 7 (19.4) | | | 3.8 (1.5, 10.0) | | 0.033 |
| **Changes in RLS-6 scores** | | | | | | | |
| **Parameter** | | | **Treatment difference:  FCM – placebo** | | | | |
|  |  |  | **LS mean** | **95% CI** | | ***P* value** | |
| Sleep satisfaction | | | 1.2 | −2.22, −0.12 | | 0.029 | |
| Severity when falling asleep | | | –1.9 | −3.04, −0.74 | | 0.002 | |
| Severity during the night | | | –1.4 | −2.44, −0.26 | | 0.016 | |
| Severity during the day (at rest) | | | –1.6 | −2.67, −0.53 | | 0.004 | |
| Severity during the day (active) | | | –0.9 | −1.79, 0.05 | | 0.064 | |
| Daytime tiredness | | | –1.5 | −2.47, −0.56 | | 0.002 | |

CGI, Clinical Global Impression; CI, confidence interval; FCM, ferric carboxymaltose; LS, least squares; PGI-I, Patient Global Impression of Improvement; RLS-6, Restless Legs Syndrome-6.

**SUPPLEMENTARY TABLE 3. Treatment-emergent adverse events overall (≥2% incidence in either treatment arm)**

| **Adverse event, n (%)*** | **Ferric carboxymaltose**  **(n = 58)** | **Placebo  (n = 52)** |
| --- | --- | --- |
| Headache | 7 (12) | 2 (4) |
| Nausea | 3 (5) | 0 |
| Gastroenteritis | 3 (5) | 1 (2) |
| Influenza | 3 (5) | 2 (4) |
| Nasopharyngitis | 3 (5) | 5 (10) |
| Arthralgia | 3 (5) | 1 (2) |
| Pyrexia | 2 (4) | 0 |
| Back pain | 2 (4) | 0 |
| Pruritus | 1 (2) | 2 (4) |
| Insomnia | 1 (2) | 2 (4) |
| Feeling cold | 1 (2) | 2 (4) |
| Abdominal pain upper | 1 (2) | 2 (4) |
| Diarrhea | 0 | 2 (4) |

*By the Medical Dictionary for Regulatory Activities preferred term.
