# Supplementary material for: Ferric carboxymaltose in patients with restless legs syndrome and nonanemic iron deficiency: A randomized trial
Source: Mov Disord. 2017 Jun 23;32(10):1478–82. doi: 10.1002/mds.27040 (PMC5655783; doi:10.1002/mds.27040)
Supplement: Supplementary file 4 — Supplementary Information [file MDS-32-1478-s004.docx]

**Ferric Carboxymaltose in Patients with Restless Legs Syndrome and Nonanemic Iron Deficiency: A Randomized Trial**

**– Supplementary Materials**

**Methods**

**Patients**

The first patient was screened on 8 April 2014 and the last subject completed the study on 1 September 2015. The inclusion criteria also specified patients either to be naïve to RLS medication or not to have taken any RLS medication for at least 7 days prior to study initiation. Medications taken prior to the start of the trial can be found in Suppl. Table 1. Patients were excluded if they had a history or presence of severe psychiatric disorder, history of severe systemic diseases or clinically relevant hepatic dysfunction, current augmentation of restless leg syndrome (RLS), acute or chronic infection, known relevant cardiac dysfunction and/or arrhythmias, known history or presence of moderate/severe pain disorders, hemoglobinopathy, hemochromatosis, or other iron-storage disorders.

Informed consent forms (ICFs) were aligned according to the ethical, regulatory, and legal requirements of the participating country. Patients were informed that their participation in the study was entirely voluntary, would have no effect on clinical care otherwise available, and that they could withdraw consent to participate at any time without penalty or loss of further medical treatment. In Germany and Finland, each patient signed two ICFs: the first was to provide informed consent for the initial serum ferritin and transferrin saturation test, once the patient was found to be eligible; a second ICF was completed prior to undergoing a full screening assessment. In Switzerland, the
two-step screening procedure was the same; however, consent to both steps was provided in one ICF.

**Study Design**

Randomization was performed based on a pre-defined randomization list, stratified per site, and generated by the Sponsor’s Biostatistics department, to which only unblinded study staff had access. Patients were allocated a randomization number in accordance with the randomization schedule generated by the Sponsor’s Biostatistics department. This number corresponded to a unique envelope containing the treatment assigned. In patients who required additional non-ferric carboxymaltose treatment due to lack or loss of efficacy before day 85 (week 12), study participation was terminated and all assessments planned for the week 12 visit were performed. The protocol was approved by the institutional review boards or independent ethics committees of all investigational sites.

**Study Design**

Other secondary efficacy endpoints included differences between treatment groups in: (1) item-1 of the Clinical Global Impression scale (CGI-Item 1); (2) CGI-Item 2 and the Patient Global Impression of Improvement Index (PGI-I); (3) responder rates, defined as (A) the proportion of patients with ≥50% improvement in IRLS, or (B) ≥6-point improvement in IRLS score; (4) day and evening symptom severity according to the RLS-6 scale; and
(5) quality of life (QoL)-RLS and the Medical Outcomes Study (MOS) sleep scale scores.

**Assessments**

The IRLS scale consisted of 10 graded questions measuring impairments of mood and daily activities.^1^ IRLS total scores of 11–20 and 21–30 denote moderate and severe RLS, respectively.^1^ Secondary efficacy assessments included the seven-point CGI severity rating scale item-1; CGI item-2 was combined with the single-question PGI-I (PGI-I assessed at baseline, and weeks 4 and 12 only).^2^ The RLS-6 rating scale assessed RLS severity during day- and night-time periods. Patients were also required to report on their QoL (QoL-RLS questionnaires),^3^ sleep satisfaction and severity of tiredness during the day (MOS sleep scale, assessed at baseline, and weeks 4 and 12 only).^3^

**Statistical Analyses**

The full analysis set comprised of all randomized patients who received study treatment and had at least one baseline and one post-baseline assessment; whilst the safety set included all randomized patients who received the study drug. The last observation carried forward method was used to impute values for patients with missing scores at weeks 4, 8, and 12. Treatments were compared by an Analysis of Covariance (ANCOVA) (treatment, baseline score and baseline serum ferritin measured as fixed effects; centers as random effect). The ANCOVA adjusted for covariables or imbalances in the treatment groups to calculate the least-squares (LS) means; *t*-tests were subsequently carried out on LS means to test for significant differences between the adjusted groups. A correlation analysis was conducted between baseline iron parameter levels and IRLS changes from baseline to all assessment weeks (observed data) using the Pearson’s correlation coefficient. Safety and tolerability analyses were conducted on the safety set, including all randomized patients who received the study drug.

**Results**

**Secondary Efficacy Endpoints**

Significantly more responders were observed with ferric carboxymaltose by end of study (EOS). Improvements of ≥50% in IRLS score were reported for 22 patients (37.3%) treated with ferric carboxymaltose versus 10 placebo patients (19.6%; *P* = 0.042; Suppl. Fig. 2a). In particular, 72.9% (n = 43) of patients treated with ferric carboxymaltose achieved an IRLS score improvement of ≥6 points at any time during treatment, compared with 47.1% (n = 24) of those receiving placebo (*P* = 0.006). Additionally, FCM treated patients achieved the greatest improvement in IRLS score, from baseline, at week 12 (n=16; Suppl. Fig 2b). In those patients who did achieve improvements in IRLS scores, half achieved their largest improvement in Weeks 1 and 4 (total n=26) and the other half in Week 8 and 12 (total n=26). Furthermore, significant improvements in CGI-Item 1 scores were observed with ferric carboxymaltose compared with placebo at week 8 (treatment difference –0.62 [95% CI –1.14, –0.09], *P* = 0.023) and week 12 (–0.70 [95% CI –1.25, –0.15], *P* = 0.013; Suppl. Fig. 2c) and the proportion of patients rating improvements in CGI-Item 2 and PGI-I scores were significantly greater with ferric carboxymaltose treatment than placebo (Suppl. Table 2). Significant improvements from baseline to EOS were in favor of ferric carboxymaltose for most categories of the RLS-6 assessments (Suppl. Table 2).

## Quality of Life and Sleep Assessments

Significant improvements in patients treated with ferric carboxymaltose compared with placebo were reported for most categories of the QoL-RLS assessments, including effect of RLS symptoms (*P* = 0.011), effects of other features (*P* = 0.001), and way of handling RLS symptoms (*P* = 0.006). Comparable improvements in mean MOS sleep scores were observed with both treatments.

**Tolerability**

The most common treatment-emergent adverse event (TEAE) in the ferric carboxymaltose group was headache (seven patients [12.1%]; Suppl. Table 3), whereas nasopharyngitis was most common in the placebo group (five patients [9.6%]; Suppl. Table 3). Severe TEAEs experienced in the FCM group were headache; insomnia; sleep attacks and road traffic accident; increase in RLS symptom severity (one patient each). Whereas arthralgia and a decrease in serum ferritin were experienced in the placebo group (one patient each).

Serious AEs were reported in one FCM and one placebo group patient. Within the ferric carboxymaltose group, a 66-year-old female patient experienced two SAEs, which were considered unrelated to the study medication but related to study design. This patient had concurrent conditions of gastritis and chronic insomnia, which had been treated with omeprazole (20 mg daily) and lorazepam (1 mg daily), respectively. The patient stopped lorazepam treatment but continued with omeprazole on the day of her screening visit. The patient did not experience any non-serious AEs during the study; on day 18, she experienced a sleep attack, which was attributed to the withdrawal of her usual medication (lorazepam) and then resulted in a road traffic accident. Subsequently, the patient was withdrawn from the study and recovered.

**References**

1. Walters AS, LeBrocq C, Dhar A *et al*. Validation of the International Restless Legs Syndrome Study Group rating scale for restless legs syndrome. *Sleep Med* 2003;4:121-132.

2. National Institute of Mental Health. 028 CGI. Clinical Global Impressions. In: Guy W, ed. *ECDEU Assessment Manual for Psychopharmacology*, pp. 217-222.1976. Rockville (MD): National Institute of Mental Health.

3. Kohnen R, Martinez-Martin P, Benes H *et al*. Rating of daytime and nighttime symptoms in RLS: validation of the RLS-6 scale of restless legs syndrome/Willis-Ekbom disease. *Sleep Med* 2016;20:116-122.

**Supplementary Table and Figure Legends**

**SUPPL. TABLE 1. Medications taken prior to trial initiation (≥2% incidence in either treatment arm)**

**SUPPL. TABLE 2. Symptom severity according to Clinical Global Impression-Item 2 and Patient Global Improvement Index scores Restless Legs Syndrome-6 scores**

**SUPPL. TABLE 3. Treatment-emergent adverse events overall (≥2% incidence in either treatment arm)**

**SUPPL. FIG. 1. Disposition of study patients.**

**SUPPL. FIG 2. (a) Responder rates: proportion of patients with an improvement of ≥50% in IRLS total score; (b) Time when FCM patients achieved the highest IRLS score improvement (c) Changes in Clinical Global Impression-Item 1 scores over time**
